# A Causal Inference Study of Circulating Metabolites Mediating the Effect of Obesity‐Related Indicators on the Incidence of Anxiety Disorders

**DOI:** 10.1002/brb3.70653

**Published:** 2025-07-07

**Authors:** Cheng Qin, Wenbin Gai

**Affiliations:** ^1^ Xijing Hospital The Fourth Military Medical University Xi'an China; ^2^ Beijing Institute of Basic Medical Sciences Beijing China

**Keywords:** AD, circulating metabolites, Mendelian randomization, obesity

## Abstract

**Background:**

Previous studies have reported an association between obesity and anxiety; however, these findings are inconclusive and subject to the risk of reverse causality.

**Methods:**

Employing data from the UK Biobank and FinnGen Consortium, this study conducted genome‐wide association studies (GWAS) and employed a two‐sample Mendelian Randomization (MR) approach to investigate the association between genetic predictors of obesity and anxiety disorders (AD). A multivariable MR (MVMR) methodology quantified the proportion of the impact of obesity‐related indicators on AD mediated by circulating metabolites, accompanied by sensitivity analyses to assess the robustness of the results.

**Results:**

A univariate MR analysis demonstrated that a higher body fat percentage correlates with an elevated risk of anxiety (OR = 1.1428; *p* = 0.033). In contrast, increased levels of obesity and hyperalimentation were linked to a decreased risk of anxiety (OR = 0.9018; *p* = 0.044). Additionally, twelve circulating metabolites were identified as causally related to AD. Reverse causation MR analysis affirmed the lack of reverse causal influence of AD on obesity markers and circulating metabolites (*p* > 0.05). MVMR analysis revealed that body fat percentage and anxiety are interconnected through mediated pathways involving phenylalanine and the ratio of free cholesterol to total lipids in very small VLDL, with mediation effects recorded at 0.028 and 0.014, respectively. Furthermore, body fat percentage impacts anxiety via the ratio of linoleic acid to total fatty acids, demonstrating a partial mediation effect of −0.042, which accounts for 25% of the overall impact. Obesity and other hyperalimentations influence anxiety through phenylalanine, with a partial mediation effect of 0.006, accounting for 7.59% of the total effect.

**Conclusion:**

This study establishes a significant causal relationship between obesity and anxiety, with three circulating metabolites playing a clear mediating role. Thus, targeting these metabolites could significantly reduce the burden of AD associated with obesity.

## Graphical abstract

This Mendelian randomization study reveals that higher body fat percentage increases anxiety disorder risk (OR = 1.14), while certain obesity phenotypes show protective effects (OR = 0.90). Three key circulating metabolites—phenylalanine, the free cholesterol to total lipids ratio in very small VLDL, and the linoleic acid ratio—mediate these relationships. Phenylalanine and free cholesterol completely mediate body fat's effect on anxiety, while the linoleic acid ratio partially mediates this relationship (25% of the total effect).

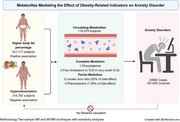

## 1 Introduction

AD is among the most common mental disorders, affecting approximately 301 million people globally (GBD 2016 Disease and Injury Incidence and Prevalence Collaborators 2017; Penninx et al. 2021). Despite advances in research, challenges persist in the diagnosis and treatment of AD (Szuhany and Simon 2022). Treatment efficacy varies across different subtypes and severities of anxiety, with a significant proportion of patients experiencing poor responses and adverse effects from medications (Ströhle et al. 2018). These issues intensify the burden on both patients and society (Javaid et al. 2023). Research into the causes and pathogenic mediators of AD is crucial, aiming at the early identification and intervention for high‐risk individuals.

Obesity, associated with various physical and psychological conditions (Avila et al. 2015; Rajan and Menon 2017), demonstrates inconsistent findings in its relationship with AD (Fulton et al. 2022). This inconsistency is largely attributed to the reliance on BMI, which does not accurately reflect true body fat content and may misrepresent obesity status, particularly in individuals with high muscle mass, such as athletes (Amiri and Behnezhad 2019; J. Wang et al. 2022; R.‐Z. Wang et al. 2024). Moreover, the infrequent use of body fat percentage—a more precise marker of obesity—in research (Etchison et al. 2011), combined with potential biases from reverse causation or confounding in observational studies, further complicates the understanding of this relationship.

It is currently believed that obesity induces AD through mechanisms such as neuroinflammation and social factors. In fact, metabolic dysregulation represents a significant pathological change during obesity. The brain, an organ requiring high metabolic input, experiences metabolic changes that could precipitate a range of mental disorders (Xiao et al. 2022). Little is known about the metabolic products that might trigger AD. Thus, it is hypothesized that obesity mediates the causal link between obesity and AD through the alteration of circulating metabolic products. Understanding the role of these products not only aids in deciphering the pathophysiological mechanisms underlying AD but also assists in identifying effective biomarkers and drug targets to refine treatment strategies.

Here, a univariable MR analysis was performed to investigate the causal relationships between obesity‐related markers, circulating metabolites, and AD. Subsequently, an MVMR analysis investigates the direct effects of circulating metabolites on anxiety, ultimately assessing the mediation effect of obesity‐related indicators on the onset of anxiety through these metabolites.

## 2 Materials and Methods

### 2.1 Study Reporting Guidelines and Study Design

**FIGURE 1:**
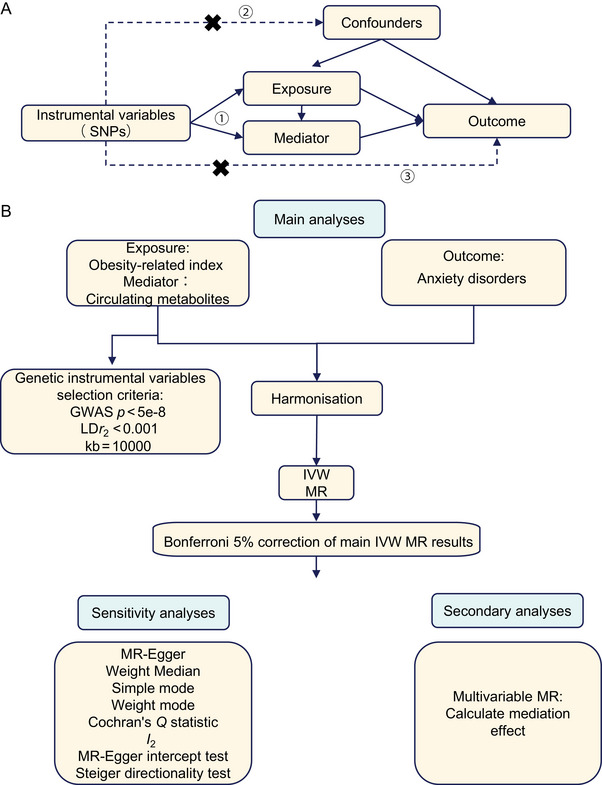
Flowchart of MR Analysis. (A) Fundamental assumptions of MR analysis: This encompasses: (1) Association Assumption: Instrumental variables must be robustly associated with the exposure variable; (2) Exclusivity Limitation: Instrumental variables should affect the outcome solely through the causal pathway: “instrumental variable → exposure → outcome.” (3) Independence Assumption: Instrumental variables should be independent of any confounders that could influence both the exposure and the outcome. (B) Analytical methods flowchart of this study.

Investigating the mediating effects of obesity‐related indices and circulating metabolites in the genesis of AD connected to obesity, this research used two‐sample MR and public datasets to examine the influence of these variables on anxiety. The technique followed the rules laid out in the STROBE‐MR Statement, which aims to improve the reporting of observational studies in epidemiology (Skrivankova et al. 2021). Figure 1 shows a schematic depicting the study's design.

### 2.2 Data Sources

GWAS data for obesity‐related indicators: Obesity and other hyperalimentation (finn‐b‐E4_OBESITY_HYPER): This GWAS analysis is based on data from 218,792 subjects of European ancestry. Body fat percentage (ukb‐a‐264): Conducted by Neale et al., this analysis involves data from 331,117 subjects (Neale, 2017). GWAS data on circulating metabolite indicators: Analysis by Bycroft et al., involving indicators from 115,078 participants (aged ≥ 18 years) of European ancestry in the UK Biobank (https://gwas.mrcieu.ac.uk/) (Bycroft et al. 2018).

AD GWAS data (finn‐b‐KRA_PSY_ANXIETY): for Finns who lived at least 18 years old, the FinnGen project combines genetic information with electronic health records. Included in this collection are hospital biobank samples, disease‐specific cohorts, and prospective epidemiological cohorts. Anxiety GWAS data includes 20,992 cases and 197,800 controls from the FinnGen Consortium GWAS analysis (Kurki et al. 2023).

### 2.3 Instrumental Variable Selection

There are three basic requirements for a genetic variation instrumental variable to be valid: (1) it needs to have a strong relationship with the exposure being studied (association hypothesis); (2) it needs to have no significant correlation with any potential confounders that could impact the exposure or outcome (independence assumption); and (3) it needs to impact the outcome solely through the chain reaction of “instrumental variable → exposure → outcome” (exclusivity limitation).

To evaluate the effect of exposure on AD, initial screening criteria for MR analysis took into account the small sample size of GWAS data for obesity indices and the exploratory character of this research. The criteria included choosing SNPs using conventional GWAS cutoffs (*p *< 5e‐8, clump = TURE, *r* ^2 ^< 0.001, kb = 10000). For reverse causality MR analysis concerning AD, SNPs were selected with a threshold of *p *< 5e‐6, excluding those in linkage disequilibrium (*r* ^2 ^< 0.001 and a physical distance exceeding 10,000 kb between genes). Instrumental variables were subsequently extracted from GWAS outcome data based on these SNPs.

### 2.4 MR Causal Effect Estimation

Inverse‐Variance Weighted (IVW), MR‐Egger, Weighted Median, simple mode, and weighted mode were among the several two‐sample MR approaches used to determine exposure‐outcome causal linkages. The IVW approach is well‐known for being robust under certain conditions (Bowden et al. 2016). When pleiotropy and heterogeneity are not present, it uses the inverse of the result variance for weighting and mostly leaves out the intercept term in regression. In cases where there is heterogeneity, the IVW random effects model is used to augment this method along with the other four approaches. The results were computed using the MR‐Egger method when pleiotropy was discovered.

### 2.5 Sensitivity Analysis

This component included various tests such as the heterogeneity test, pleiotropy test, and leave‐one‐out test:

Heterogeneity test: A statistically significant result was obtained by applying the Cochran *Q* test to assess heterogeneity among SNP estimations, hence demonstrating high heterogeneity. This test confirms that heterogeneity exists, but it cannot tell you where it is distributed. Consequently, the *I* ^2^ statistic was used to gauge the extent of heterogeneity relative to total variation. Interpretations of the *I* ^2^ values were as follows: *I* ^2 ^≤ 0 was set to 0, indicating no observed heterogeneity; *I* ^2 ^= 0%–25% indicated mild heterogeneity; *I* ^2 ^= 25%–50% indicated moderate heterogeneity; and *I* ^2 ^> 50% indicated high heterogeneity. The formula for *I* ^2^ is as follows:

$$I^{2}=\frac{Q-\mathrm{df}}{Q}=\times 100\%$$

(2) Pleiotropy test: The MR‐Egger method assessed instrumental variable pleiotropy. A significant intercept (*p* < 0.05) suggested notable horizontal pleiotropy.

(3) Leave‐one‐out test: To evaluate how well the MR estimations held up against each SNP, we methodically removed them one by one. If there were large differences in the MR effect estimates when a particular instrumental variable was included and when it was not, it meant that the MR effect was sensitive to that SNP.

### 2.6 Multivariate MR Analysis and Mediating Effect Estimation

MVMR is an expansion of classic MR that estimates the impact of many exposures on a single outcome by using genetic variants connected to multiple related exposures. The direct impacts of exposures on individuals are determined using this method. In this study, the indirect pathways from obesity‐related indicators through circulating metabolites to AD were explored using univariate MR to establish the effects of obesity indicators on metabolites (Figure 1). Mediating effects were quantified using the following formula:

$$\beta_{M}=\beta_{A}\;\times \;\beta_{B}$$

$$SE_{M}=\sqrt{{\left(\beta_{A}\times SE_{B}\right)}^{2}+{\left(\beta_{B}\times SE_{A}\right)}^{2}+S{E_{A}}^{2}\times S{E_{B}}^{2}}$$

*β_M_* is the MR effect value of obesity‐related indicators on circulating metabolites, and *β_A_* represents the MR effect value of obesity‐related indicators on circulating metabolites. *β_B_* denotes the direct effect value of circulating metabolites on AD, as obtained by multivariate MR; SE*_M_* is the standard error of mediating effect; SE*_A_* is the standard error of MR analysis for obesity‐related indicators on circulating metabolites; and SE*_B_* is the standard error of MR analysis for circulating metabolites on AD. If *β_M_* is significantly associated with obesity and the MR effect of anxiety shows an opposite sign to *β_C′_*, the proportion of the direct effect mediated by circulating metabolites is calculated as |*β_M_*/*β_C′_* | × 100%. Conversely, if *β_M_* is significantly associated with obesity and the MR effect of anxiety on the value of *β_C′_*, the proportion of mediation by circulating metabolites relative to the total effect is calculated as *β_M_*/*β_C _* × 100%, where *β_C_* represents the MR effect value of the anxiety disorder‐related index on obesity. Owing to the complexity of mediating effects, this paper focuses on cases where both the exposure mediators and the outcome show significant cause‐and‐effect relationships and a significant causal correlation exists between them.

### 2.7 Statistical Analysis

All data analyses were conducted using R (version 4.2.2). The TwoSampleMR package was implemented for MR analyses (Hemani et al. 2017). The robustness and reliability of results were verified through Cochran *Q* tests and leave‐one‐out analysis. Genetic pleiotropy was assessed using the MR‐Egger intercept method, while the Steiger directivity test within the TwoSampleMR package was used to establish causality direction. In the MR analysis, odds ratios (OR) and 95% confidence intervals (95% CI) were used as metrics, with *p* < 0.05 denoting statistical significance.

## 3 Results

### 3.1 Instrumental Variable Screening

Genetic variants showing evidence of linkage disequilibrium were not included in this analysis because they did not meet the criteria for instrumental variables. Once the GWAS data for AD had been matched, SNPs linked to markers of obesity and circulating metabolites could be included as instrumental variables. In Table S1, we can see how many instrumental variables were used for each indicator, which shows which indicators were significant in the MR analysis (*p* < 0.05).

### 3.2 MR Causal Effect Estimates

**FIGURE 2:**
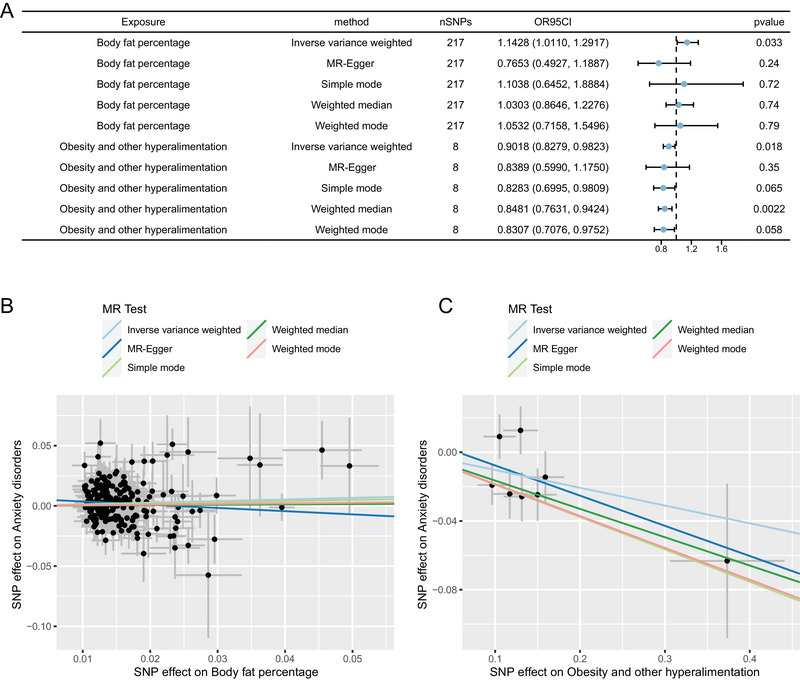
Multiple model analysis results and effect estimates for the MR analysis of obesity‐related indicators and AD. (A) Forest plots display the causal association results from various MR models concerning obesity‐related indicators and AD. Each model's number of instrumental variables, along with beta values and standard errors, is depicted. (B, C) Scatter plots illustrate the causal relationships between body fat percentage (B) and obesity and other hyperalimentation (C) with AD. The lines’ slopes represent the magnitude of the causal relationships as predicted by different models.

Five models, including MR Egger, Weighted Median, IVW, SM, and Weighted Mode, were utilized for analysis. The IVW model results for the effect of obesity‐related indicators on anxiety (Figure 2A) demonstrated that both obesity and other hyperalimentation (finn‐b‐E4_OBESITY_HYPER) and body fat percentage (ukb‐a‐264) are significantly associated with AD. Specifically, an increase in body fat percentage (OR = 1.1428; *p* = 0.033) corresponds to a higher risk of anxiety, whereas an increase in obesity and other hyperalimentation (OR = 0.9018, *p* = 0.018) is associated with a lower risk of anxiety. Consistent directional estimates and slopes were observed across different models for obesity and other hyperalimentation (Figure 2B) and body fat percentage (Figure 2C).

The results of the IVW model for circulating metabolites on AD are displayed in Figure S1. Significant causal associations were found between various ratios of cholesterol and other lipids in different VLDL sizes and AD. These include:

1. Cholesterol to total lipids ratio (TLR) in medium VLDL (met‐d‐M_VLDL_C_pct, OR = 0.909, *p* = 0.042), small VLDL (met‐d‐S_VLDL_C_pct, OR = 0.915, *p* = 0.022), and very small VLDL (met‐d‐XS_VLDL_C_pct, OR = 0.923, *p* = 0.048).
2. Cholesteryl esters to TLR in medium VLDL (met‐d‐M_VLDL_CE_pct, OR = 0.901, *p* = 0.038).
3. Degree of unsaturation (met‐d‐Unsaturation, OR = 0.936, *p* = 0.035).
4. Free cholesterol to TLR in medium VLDL (met‐d‐M_VLDL_FC_pct, OR = 0.911, *p* = 0.032) and very small VLDL (met‐d‐XS_VLDL_FC_pct, OR = 0.903, *p* = 0.020).
5. Phenylalanine (met‐d‐Phe, OR = 1.227, *p* = 0.049).
6. Ratio of linoleic acid (LA) to total fatty acids (met‐d‐LA_pct, OR = 1.122, *p* = 0.023).
7. Triglycerides to TLR in medium VLDL (met‐d‐M_VLDL_TG_pct, OR = 1.104, *p* = 0.039), small VLDL (met‐d‐S_VLDL_TG_pct, OR = 1.102, *p* = 0.016), and very small VLDL (met‐d‐XS_VLDL_TG_pct, OR = 1.110, *p* = 0.0099).

The MR analysis of five models of circulating metabolites influencing AD is depicted in Figure S2A–L. The scatter plot fitting curves of these models are largely consistent, with most slopes showing uniformity, and the intercept of the IVW model is nearly zero, indicating a close alignment with the null hypothesis.

### 3.3 Sensitivity Analysis

**FIGURE 3:**
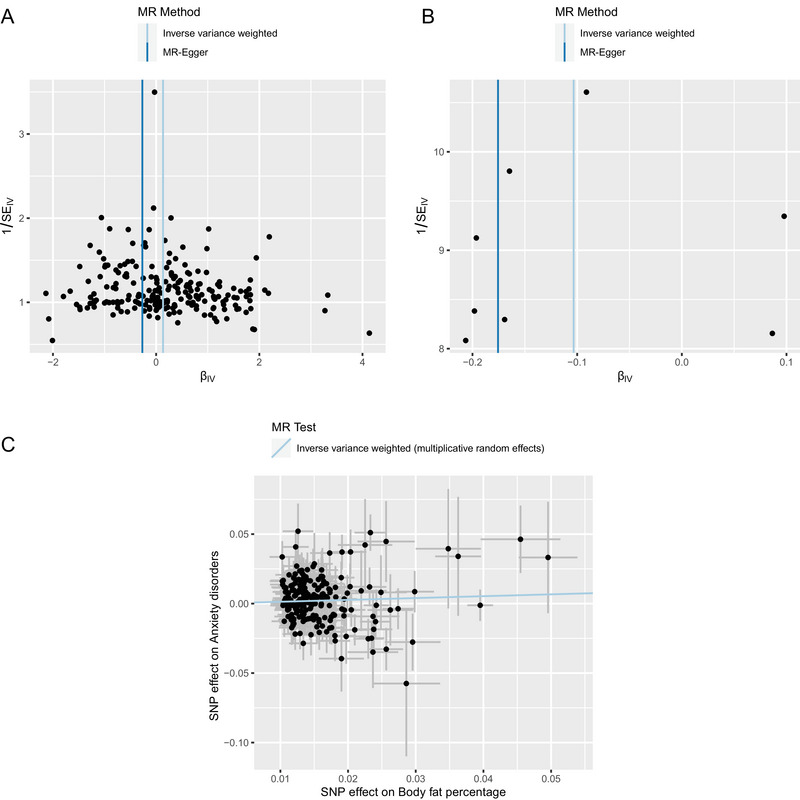
MR analysis heterogeneity test funnel plot and IVW random effects model effect estimates for obesity‐related indicators and anxiety disorder. (A–B) Funnel plots display causal effect estimates for each instrumental variable concerning body fat percentage (A) and obesity and other hyperalimentation (B) with AD. The IVW and MR‐Egger models provided causal effect estimates graphically represented on plots. (C) A scatter plot delineated the causal relationship between body fat percentage and AD, where the slope of the line, estimated by the IVW random effects model, quantified the causal interaction.

Significant results from MR analysis of obesity‐related indicators, such as body fat percentage (id:ukb‐a‐264), exhibited high heterogeneity (*p* < 0.05) and *I* ^2^ statistic, detailed in Table S2. The symmetry observed in the funnel plots for body fat percentage (Figure 3A) and obesity, along with other hyperalimentation factors (Figure 3B), suggests a lack of potential bias in the estimated effects of causal associations. For these indicators showing considerable heterogeneity, the random effects model within the IVW framework was utilized for causal estimation (Figure 3C, Table S3 **)**.

The instrumental variables were subjected to MR‐Egger regression in order to assess the possible impact of horizontal pleiotropy. It was confirmed that the causal evaluations were unaffected by horizontal pleiotropy by looking at the intercept terms for each index, which showed *p* values better than 0.05 and intercepts approaching 0 (Table S4).

A sensitivity analysis employing sequential exclusion tests indicated consistent effect estimates for metrics associated with obesity, thus validating the robustness of these findings (Table S5).

Results from the reverse causality MR analysis demonstrated that anxiety did not exert a causal impact on crucial obesity‐related metrics (*p* > 0.05) (Table S6).

Table S7 details the use of the Cochran *Q* test and the *I* ^2^ statistic to quantify the heterogeneity of significant findings. The ratios of cholesterol to total lipids, cholesteryl esters to total lipids, free cholesterol to total lipids, and triglycerides to total lipids in medium VLDL (id:met‐d‐M_VLDL_TG_pct) showed significant heterogeneity (*p* < 0.05). Funnel plots for diverse metabolites displayed a symmetrical distribution of causal association effects, indicating an absence of bias in these results (Figure S3A–L). The causal influences were estimated using the random effects model of IVW for measures that showed significant heterogeneity (Figure S4, Table S8).

Applying MR‐Egger regression again to measure horizontal pleiotropy in instrumental variables revealed that the intercepts were close to zero and the *p* values for intercept terms were more than 0.05, proving that horizontal pleiotropy did not affect the causal inferences (Table S9).

A leave‐one‐out sensitivity analysis underscored stable effect estimates for obesity‐linked measures, reinforcing the reliability of these outcomes (Table S10).

Furthermore, reverse causality MR analysis underscored that anxiety did not causally affect significant circulating metabolites (*p* > 0.05) (Table S11).

### 3.4 Multivariate MR Analysis

Before conducting the MVMR analysis, MR analyses were performed on obesity‐related indicators and circulating metabolites with significant causal effects on AD, and multivariate MR exposure factors were screened. Significant causal effects were noted for certain circulating metabolites on body fat percentage and for phenylalanine on obesity and other hyperalimentation (Table S12). Steiger directivity tests confirmed no reverse causal effects (Table S13).

Consequently, these impactful findings were utilized as the basis for individual multivariate MR analyses focusing on AD (Table 1). Model 1, incorporating body fat percentage and the ratio of circulating metabolites of LA to total fatty acids, demonstrated significant associations with anxiety (*p* < 0.05). Conversely, Model 2, which integrated body fat percentage with the cholesterol to TLR in medium VLDL, did not reveal any significant associations with anxiety (*p* > 0.05). Similarly, Model 3, examining the relationship between body fat percentage and cholesteryl esters to TLR in medium VLDL, also failed to show significant effects on anxiety (*p* > 0.05).

**TABLE 1:** MVMR analysis of the effects of obesity‐related index and circulating metabolites on AD.

Model	Exposure	Outcome	nSNP	Beta	SE	*p* value
Model1	Ratio of linoleic acid to total fatty acids	AD	22	0.107997	0.048853	0.027059
	Body fat percentage	AD	211	0.167564	0.064686	0.009586
Model2	Cholesterol to TLR in medium VLDL	AD	50	−0.05973	0.034843	0.086501
	Body fat percentage	AD	193	0.080173	0.063223	0.204761
Model3	Cholesteryl esters to TLR in medium VLDL	AD	48	−0.06762	0.035611	0.057577
	Body fat percentage	AD	196	0.084584	0.063718	0.184348
Model4	Free cholesterol to TLR in medium VLDL	AD	45	−0.06296	0.035103	0.072882
	Body fat percentage	AD	192	0.071947	0.063899	0.260182
Model5	Triglycerides to TLR in medium VLDL	AD	45	0.05389	0.035401	0.127945
	Body fat percentage	AD	195	0.088134	0.062745	0.160128
Model6	Phenylalanine	AD	5	0.166185	0.082412	0.043747
	Body fat percentage	AD	227	0.075594	0.061702	0.220515
Model7	Cholesterol to TLR in small VLDL	AD	43	−0.05207	0.034527	0.131504
	Body fat percentage	AD	195	0.105199	0.063652	0.098386
Model8	Triglycerides to TLR in small VLDL	AD	42	0.065161	0.035899	0.069508
	Body fat percentage	AD	197	0.082684	0.062357	0.184849
Model9	Degree of unsaturation	AD	32	−0.05806	0.029936	0.05244
	Body fat percentage	AD	211	0.096866	0.061906	0.117651
Model10	Cholesterol to TLR in very small VLDL	AD	47	−0.04066	0.033698	0.227533
	Body fat percentage	AD	196	0.096231	0.064351	0.134811
Model11	Free cholesterol to TLR in very small VLDL	AD	30	−0.08759	0.041249	0.033711
	Body fat percentage	AD	203	0.099101	0.065099	0.127929
Model12	Triglycerides to TLR in very small VLDL	AD	50	0.064494	0.033165	0.051819
	Body fat percentage	AD	193	0.06862	0.064037	0.283909
Model13	Phenylalanine	AD	6	0.196938	0.098572	0.045726
	Obesity and other hyperalimentation	AD	9	−0.07882	0.036252	0.029688

Model 4 assessed the impact of body fat percentage alongside the free cholesterol to TLR in medium VLDL, also yielding no significant associations with anxiety (*p* > 0.05). Model 5, examining the triglycerides to TLR in medium VLDL together with body fat percentage, similarly found no significant influence on anxiety (*p* > 0.05). In contrast, Model 6, which analyzed body fat percentage and phenylalanine, indicated a significant association for phenylalanine but not for body fat percentage regarding anxiety outcomes (*p* < 0.05 for phenylalanine and *p* > 0.05 for body fat percentage).

Additionally, Model 7, which includes body fat percentage and the cholesterol to TLR in small VLDL, along with Model 8, which combines body fat percentage with the triglycerides to TLR in small VLDL, both showed no significant effect on anxiety (*p* > 0.05). Model 9, which included body fat percentage and the degree of unsaturation of circulating metabolites, similarly indicated no significant effects on anxiety (*p* > 0.05).

Subsequent examination in Model 10, which included body fat percentage and the cholesterol to TLR in very small VLDL, as well as Model 12, which integrated body fat percentage and the triglycerides to TLR in very small VLDL, similarly yielded no significant correlations with anxiety (*p* > 0.05). Model 11, incorporating body fat percentage and the free cholesterol to TLR in very small VLDL, demonstrated a noteworthy influence of the free cholesterol to TLR on anxiety (*p* < 0.05).

Model 13, which included obesity and other hyperalimentation and phenylalanine, found significant effects for both exposures on anxiety (*p* < 0.05).

### 3.5 Mediation Effect Analysis

According to the results of multivariate analysis, significant effects on anxiety disorder were observed only in circulating metabolites in Models 1, 6, 11, and 13 (*p *< 0.05), whereas other models showed no significant effects (*p *> 0.05). Therefore, the discussion was limited to Models 1, 6, 11, and 13.

In Model 6, as body fat percentage showed no significant effect on anxiety disorder (*p *> 0.05), it was determined that body fat percentage affected anxiety disorder through the complete mediation of circulating metabolite phenylalanine. In Model 11, with no significant direct effect of body fat percentage on anxiety (*p *> 0.05), it could be inferred that body fat percentage influences anxiety through the circulating metabolite‐free cholesterol to TLR in very small VLDL, constituting a complete mediation model.

Mediation effects were estimated by comparing causal effects from univariate MR analysis with direct effects derived from multivariate MR analysis. Notably, body fat percentage mediated the effect on anxiety via the circulating metabolite phenylalanine with a mediation effect of 0.028 and via the circulating metabolite free cholesterol to TLR in very small VLDL with an effect of 0.014 (Table 2).

**TABLE 2:** Evaluation of Mendelian randomized mediating effects of the circulatory metabolism‐mediated obesity‐related index in AD (perfect mediation effect).

Exposure	Mediator	Outcomes	Total effect	Effect E–M	Effect M–O	Effect E–O	Mediation effect
Body fat percentage	Phenylalanine	AD	0.133 (0.011, 0.255)	0.168 (0.123, 0.213)	0.166 (0.005, 0.327)	0.076 (−0.046, 0.198)	0.028(0, 0.056)
Body fat percentage	Free cholesterol to TLR in very small VLDL	AD	0.133 (0.011, 0.255)	−0.155 (−0.224, −0.086)	−0.088 (−0.168, −0.008)	0.099 (−0.028, 0.226)	0.014 (0, 0.028)

In Model 1, where body fat percentage significantly influenced anxiety (*p* < 0.05), it was established that this variable mediates anxiety through the ratio of LA to total fatty acids, illustrating a partial mediation model. Similarly, Model 13 demonstrated that obesity and related hyperalimentation conditions significantly impacted anxiety (*p* < 0.05), mediated by the circulating metabolite phenylalanine, confirming another partial mediation model.

According to the details in Table 3, the mediation effect of body fat percentage on anxiety via the ratio of LA to total fatty acids was −0.042, accounting for 25% of the total effect (|−0.042/0.168 × 100%). The mediation effect of obesity and other hyperalimentation on anxiety through phenylalanine was 0.006, corresponding to 7.59% of the total effect (|0.006/−0.079| × 100%).

**TABLE 3:** Evaluation of Mendelian randomized mediating effects of the circulatory metabolism‐mediated obesity‐related index in AD (partial mediation effect).

Exposure	Mediator	Outcomes	Total effect	Effect E–M	Effect M–O	Effect E–O	Mediation effect
Body fat percentage	Ratio of linoleic acid to total fatty acids	AD	0.133 (0.011, 0.255)	−0.392 (−0.472, −0.312)	0.108 (0.012, 0.204)	0.168 (0.041, 0.295)	−0.042 (−0.081, −0.003)
Obesity and other hyperalimentation	Phenylalanine	AD	−0.103 (−0.189, −0.017)	0.03 (0.003, 0.057)	0.197 (0.003, 0.391)	−0.079 (−0.15, −0.008)	0.006 (−0.002, 0.014)

## 4 Discussion

Despite a wealth of studies examining the link between obesity and anxiety, conclusions remain disputed. Epidemiological research in European and American populations generally reveals only a weak association between obesity and AD, without significant differences (Bruffaerts et al. 2008; Hach et al. 2007; Herpertz et al. 2006; Simon et al. 2006; Zhao et al. 2009). Hughes et al. (2022) underscored the absence of evidence supporting obesity as a cause of AD in children, particularly noting the robustness of their MR study over prior observational studies. Conversely, another MR study involving over 50,000 participants suggested that obesity might alleviate anxiety (Lawlor et al. 2011). Recent studies, however, suggest that obesity may promote the onset of AD (Clark et al. 2022; R.‐Z. Wang et al. 2024; Xia et al. 2021).

Our analysis points to inconsistent definitions of obesity as a significant contributor to these conflicting findings; BMI ranges of 24–28 and above 28 are typically used to classify overweight and obesity, respectively, yet distinctions are often blurred in research (Luo et al. 2023). BMI's rapid assessment at the population level is compromised by its failure to differentiate among body fat, muscle, and bone mass, which may misclassify muscular individuals as overweight or obese, and overlooks crucial body fat distribution—a key factor in assessing risks for cardiovascular and metabolic syndromes (Etchison et al. 2011; Gallagher et al. 2000). The prevalent reliance on BMI to investigate obesity‐related AD leads to inconsistent results, rooted in BMI's inadequacy in accurately reflecting body fat percentage (Gariepy et al. 2010; Gaskell et al. 2023; J. Wang et al. 2022; Luo et al. 2023; R.‐Z. Wang et al. 2024).

In our results, we observed a paradoxical finding: body fat percentage positively correlated with anxiety risk, while obesity/hyperalimentation indicators showed a negative correlation. This apparent contradiction can be attributed to three key factors. First, methodological differences exist between the continuous physiological measurement of body fat percentage in UK Biobank versus the ICD‐coded clinical diagnoses in FinnGen. Second, certain obesity phenotypes may confer protective effects through increased serotonin precursor availability or more favorable metabolic profiles with lower inflammation. Third, our mediation analysis revealed distinct metabolic pathways—phenylalanine mediates obesity/overfeeding effects, while the LA ratio mediates body fat percentage effects. In conclusion, this paradox strengthens our findings by highlighting that obesity–anxiety relationships are mediated by distinct metabolic signatures with potentially opposing effects, underscoring the importance of personalized approaches to mental health in different obesity phenotypes.

The interplay between obesity and anxiety presents a multifaceted scenario; certain studies indicate that obesity could serve as both a precursor and a consequence of AD (Luo et al. 2023; Mulugeta et al. 2019), while alternative research suggests that persistent anxiety and associated muscle tension, commonly seen in these disorders, might result in weight loss rather than an increase in weight (Hasler et al. 2004). Observational studies may also observe transient weight fluctuations due to acute anxiety symptoms, which can obscure the long‐term relationship between anxiety and weight gain (Gariepy et al. 2010). Additionally, commonly prescribed medications for AD, including tricyclic antidepressants and selective serotonin reuptake inhibitors (SSRIs), may alter body weight, potentially causing either weight gain or loss, thereby affecting the outcomes of studies related to anxiety and obesity (Fava 2000; Lawlor et al. 2011). Our reverse causal MR analysis confirmed that anxiety does not causally affect obesity‐related indicators.

The etiology of obesity‐induced AD involves several mechanisms: chronic obesity‐related inflammation, marked by elevated levels of C‐reactive protein and interleukin‐6, may influence brain function and mood (R.‐Z. Wang et al. 2024); hormonal imbalances involving cortisol, thyroid, and sex hormones could link obesity to anxiety (Luo et al. 2023; Santiago Santana et al. 2021); obesity‐associated insulin resistance and Type 2 diabetes are implicated in anxiety through altered brain energy metabolism (Hamer et al. 2019); societal stigma and self‐perception issues may exacerbate anxiety risks in obese individuals (Puhl and Heuer 2009; Sarwer and Polonsky 2016); lifestyle factors, such as diet and physical activity, influence brain neurotransmitter balance, affecting anxiety (Jacka et al. 2010; Pedersen and Saltin 2015); and genetic/epigenetic factors (Locke et al. 2015; Wu et al. 2018), along with changes in central nervous system structure due to obesity, may contribute to anxiety pathology (Ogrodnik et al. 2019; Samodien and Chellan 2021).

This research has pinpointed three metabolic mediators—free cholesterol to TLR in very small VLDL, phenylalanine, and the ratio of LA to total fatty acids—which forge links between obesity and AD, thus paving the way for novel research avenues and therapeutic strategies. Panayotis and collegues used bioinformatics to screen cholesterol‐lowering plant sterol β‐sitosterol as an antianxiety drug candidate. Administration of β‐sitosterol alone was able to reduce stress‐induced anxiety behaviors in mice and demonstrated synergistic sedative effects when used in combination with low‐dose SSRI antidepressants (Panayotis et al. 2021). This suggests that reducing this metabolite level may help alleviate anxiety. Abnormal phenylalanine metabolism may affect neurotransmitter levels in the brain, thereby influencing mood and anxiety (Humer et al. 2020). A study focusing on PKU adolescents demonstrated that greater fluctuations and higher average levels of Phe concentration in blood corresponded to higher anxiety scores; maintaining long‐term stability of Phe significantly reduced anxiety risk. In this special population, a strict low‐phenylalanine diet has been proven to prevent and alleviate neuropsychological issues, including anxiety (Didycz and Bik‐Multanowski 2018). LA is a major ω‐6 polyunsaturated fatty acid comprising a substantial proportion of dietary fatty acids. Elevated LA levels (high ratio relative to total fatty acids or ω‐3) have been demonstrated to directly increase anxiety behaviors in animals (Clouard et al. 2015; Sakayori et al. 2016). Furthermore, epidemiological studies have found that populations with high dietary LA intake exhibit higher rates of depression and psychological distress (PMID: 35600820). Dietary interventions to reduce the LA ratio (by supplementing ω‐3 fatty acids) have shown considerable antianxiety effects (Mulugeta et al. 2019).

However, limitations include the absence of SNP data for anxiety subtypes, which raises questions about the uniformity of obesity's impact across these subtypes, and the reliance on European genetic variation data, which may not be generalizable globally. Notably, we observed discrepancies in significance levels between IVW and other MR models, which can be attributed to methodological differences. The IVW method, while statistically powerful, assumes all SNPs are valid instrumental variables, whereas methods like weighted median and MR‐Egger are more robust to potential horizontal pleiotropy by accommodating outlier effects. Although MR‐Egger tests did not detect significant horizontal pleiotropy, the conservative results from alternative methods compared to IVW's significant associations suggest underlying methodological sensitivity. These statistical uncertainties highlight that while our IVW results indicate potential associations, they require validation in larger GWAS datasets and experimental studies before drawing definitive conclusions about true biological relationships. Future research aims to experimentally confirm the mediating role of metabolic products and elucidate their mechanisms of action.

## 5 Conclusion

Our MR study clarifies the causal relationships between obesity and AD by pinpointing crucial metabolic products that act as mediators. This finding paves the way for further investigation and therapeutic approaches, underscoring the significance of metabolic regulation in the treatment of AD associated with obesity.

## Author Contributions

**Cheng Qin**: conceptualization, investigation, methodology, validation, visualization, software, formal analysis, data curation, writing – original draft. **Wenbin Gai**: supervision, writing – review and editing.

## Conflicts of Interest

The authors declare no conflicts of interest.

## Peer Review

The peer review history for this article is available at https://publons.com/publon/10.1002/brb3.70653

## Supporting information




**Supplementary Figure**: brb370653‐sup‐0001‐FigureS1.pdf

**Supplementary Figure**: brb370653‐sup‐0002‐FigureS2.pdf

**Supplementary Figure**: brb370653‐sup‐0003‐FigureS3.pdf

**Supplementary Figure**: brb370653‐sup‐0004‐FigureS4.pdf

**Supplementary Figure**: brb370653‐sup‐0005‐Table1.docx

**Supplementary Figure**: brb370653‐sup‐0006‐Table2.docx

**Supplementary Figure**: brb370653‐sup‐0007‐Table3.docx

**Supplementary Figure**: brb370653‐sup‐0008‐Table4.docx

**Supplementary Figure**: brb370653‐sup‐0009‐Table5.csv

**Supplementary Figure**: brb370653‐sup‐00010‐Table5.xlsx

**Supplementary Figure**: brb370653‐sup‐00011‐Table6.docx

**Supplementary Figure**: brb370653‐sup‐00012‐Table7.docx

**Supplementary Figure**: brb370653‐sup‐00013‐Table8.docx

**Supplementary Figure**: brb370653‐sup‐00014‐Table9.docx

**Supplementary Figure**: brb370653‐sup‐00015‐Table10.csv

**Supplementary Figure**: brb370653‐sup‐00016‐Table10.xlsx

**Supplementary Figure**: brb370653‐sup‐00017‐Table11.docx

**Supplementary Figure**: brb370653‐sup‐00018‐Table12.docx

**Supplementary Figure**: brb370653‐sup‐00019‐Table13.docx

## Data Availability

The datasets analyzed during the current study are available in the FinnGen Consortium [https://www.finngen.fi/fi] and the UK Biobank [https://www.ukbiobank.ac.uk/]. All the data generated by the MR analysis are available within the paper and its additional files.

## References

[brb370653-bib-0001] Amiri, S. , and S. Behnezhad . 2019. “Obesity and Anxiety Symptoms: A Systematic Review and Meta‐Analysis.” Neuropsychiatrie 33, no. 2: 72–89. 30778841 10.1007/s40211-019-0302-9

[brb370653-bib-0002] Avila, C. , A. C. Holloway , M. K. Hahn , et al. 2015. “An Overview of Links Between Obesity and Mental Health.” Current Obesity Reports 4, no. 3: 303–310. 26627487 10.1007/s13679-015-0164-9

[brb370653-bib-0003] Bowden, J. , G. Davey Smith , P. C. Haycock , and S. Burgess . 2016. “Consistent Estimation in Mendelian Randomization With Some Invalid Instruments Using a Weighted Median Estimator.” Genetic Epidemiology 40, no. 4: 304–314. 27061298 10.1002/gepi.21965 PMC4849733

[brb370653-bib-0004] Bruffaerts, R. , K. Demyttenaere , G. Vilagut , et al. 2008. “The Relation Between Body Mass Index, Mental Health, and Functional Disability: A European Population Perspective.” Canadian Journal of Psychiatry 53, no. 10: 679–688. 18940036 10.1177/070674370805301007

[brb370653-bib-0005] Bycroft, C. , C. Freeman, D. Petkova, et al. 2018. “The UK Biobank Resource With Deep Phenotyping and Genomic Data.” Nature 562, no. 7726: 203–209. 30305743 10.1038/s41586-018-0579-z PMC6786975

[brb370653-bib-0006] Clark, T. D. , A. J. Crean , and A. M. Senior . 2022. “Obesogenic Diets Induce Anxiety in Rodents: A Systematic Review and Meta‐Analysis.” Obesity Reviews 23, no. 3: e13399. 34811885 10.1111/obr.13399

[brb370653-bib-0007] Clouard, C. , W. J. Gerrits , I. Van Kerkhof , W. Smink , and J. E. Bolhuis . 2015. “Dietary Linoleic and α‐Linolenic Acids Affect Anxiety‐Related Responses and Exploratory Activity in Growing Pigs.” Journal of Nutrition 145, no. 2: 358–364. 25644359 10.3945/jn.114.199448

[brb370653-bib-0008] Didycz, B. , and M. Bik‐Multanowski . 2018. “Blood Phenylalanine Instability Strongly Correlates With Anxiety in Phenylketonuria.” Molecular Genetics and Metabolism Reports 14: 80–82. 29326880 10.1016/j.ymgmr.2017.12.003 PMC5758931

[brb370653-bib-0009] Etchison, W. C. , E. A. Bloodgood , C. P. Minton , et al. 2011. “Body Mass Index and Percentage of Body Fat as Indicators for Obesity in an Adolescent Athletic Population.” Sports Health 3, no. 3: 249–252. 23016014 10.1177/1941738111404655 PMC3445161

[brb370653-bib-0010] Fava, M. 2000. “Weight Gain and Antidepressants.” Supplement, Journal of Clinical Psychiatry 61, no. S11: 37–41. 10926053

[brb370653-bib-0011] Fulton, S. , L. Décarie‐Spain , X. Fioramonti, B. Guiard,, and S. Nakajima . 2022. “The Menace of Obesity to Depression and Anxiety Prevalence.” Trends in Endocrinology and Metabolism 33, no. 1: 18–35. 34750064 10.1016/j.tem.2021.10.005

[brb370653-bib-0012] Gallagher, D. , S. B. Heymsfield , M. Heo , S. A. Jebb , P. R. Murgatroyd , and Y. Sakamoto . 2000. “Healthy Percentage Body Fat Ranges: An Approach for Developing Guidelines Based on Body Mass Index.” American Journal of Clinical Nutrition 72, no. 3: 694–701. 10966886 10.1093/ajcn/72.3.694

[brb370653-bib-0013] Gariepy, G. , D. Nitka , and N. Schmitz . 2010. “The Association Between Obesity and Anxiety Disorders in the Population: A Systematic Review and Meta‐Analysis.” International Journal of Obesity 34, no. 3: 407–419. 19997072 10.1038/ijo.2009.252

[brb370653-bib-0014] Gaskell, C. , P. Sarada , E. Aleem , and G. Bendriss . 2023. “Identifying Lifestyle Factors Associated to Co‐Morbidity of Obesity and Psychiatric Disorders, a Pilot Study.” Frontiers in Public Health 11: 1132994. 37206863 10.3389/fpubh.2023.1132994 PMC10188954

[brb370653-bib-0015] GBD 2016 Disease and Injury Incidence and Prevalence Collaborators . 2017. “Global, Regional, and National Incidence, Prevalence, and Years Lived With Disability for 328 Diseases and Injuries for 195 Countries, 1990–2016: A Systematic Analysis for the Global Burden of Disease Study 2016.” Lancet 390, no. 10100: 1211–1259. 28919117 10.1016/S0140-6736(17)32154-2 PMC5605509

[brb370653-bib-0016] Hach, I. , U. E. Ruhl , M. Klose , J. Klotsche , W. Kirch , and F. Jacobi . 2007. “Obesity and the Risk for Mental Disorders in a Representative German Adult Sample.” European Journal of Public Health 17, no. 3: 297–305. 16973642 10.1093/eurpub/ckl227

[brb370653-bib-0017] Hamer, J. A. , D. Testani , R. B. Mansur , Y. Lee , M. Subramaniapillai , and R. S. McIntyre . 2019. “Brain Insulin Resistance: A Treatment Target for Cognitive Impairment and Anhedonia in Depression.” Experimental Neurology 315: 1–8. 30695707 10.1016/j.expneurol.2019.01.016

[brb370653-bib-0018] Hasler, G. , D. S. Pine , A. Gamma , et al. 2004. “The Associations Between Psychopathology and Being Overweight: A 20‐Year Prospective Study.” Psychological Medicine 34, no. 6: 1047–1057. 15554575 10.1017/s0033291703001697

[brb370653-bib-0019] Hemani, G. , K. Tilling , and G. Davey Smith . 2017. “Orienting the Causal Relationship Between Imprecisely Measured Traits Using GWAS Summary Data.” PLos Genetics 13, no. 11: e1007081. 29149188 10.1371/journal.pgen.1007081 PMC5711033

[brb370653-bib-0020] Herpertz, S. , R. Burgmer , A. Stang , et al. 2006. “Prevalence of Mental Disorders in Normal‐Weight and Obese Individuals With and Without Weight Loss Treatment in a German Urban Population.” Journal of Psychosomatic Research 61, no. 1: 95–103. 16813851 10.1016/j.jpsychores.2005.10.003

[brb370653-bib-0050] http://www.nealelab.is/blog/2017/7/19/rapid‐gwas‐of‐thousands‐of‐phenotypes‐for‐337000‐samples‐in‐the‐uk‐biobank.

[brb370653-bib-0021] Hughes, A. M. , E. Sanderson , T. Morris , et al. 2022. “Body Mass Index and Childhood Symptoms of Depression, Anxiety, and Attention‐Deficit Hyperactivity Disorder: A Within‐Family Mendelian Randomization Study.” eLife 11: e74320. 36537070 10.7554/eLife.74320 PMC9767454

[brb370653-bib-0022] Humer, E. , C. Pieh , and T. Probst . 2020. “Metabolomic Biomarkers in Anxiety Disorders.” International Journal of Molecular Sciences 21, no. 13: 4784. 32640734 10.3390/ijms21134784 PMC7369790

[brb370653-bib-0023] Jacka, F. N. , J. A. Pasco , A. Mykletun , et al. 2010. “Association of Western and Traditional Diets With Depression and Anxiety in Women.” American Journal of Psychiatry 167, no. 3: 305–311. 20048020 10.1176/appi.ajp.2009.09060881

[brb370653-bib-0024] Javaid, S. F. , I. J. Hashim , M. J. Hashim , E. Stip , M. A. Samad , and A. A. Ahbabi . 2023. “Epidemiology of Anxiety Disorders: Global Burden and Sociodemographic Associations.” Middle East Current Psychiatry 30, no. 1: 44.

[brb370653-bib-0025] Kurki, M. I. , J. Karjalainen, P. Palta, et al. 2023. “FinnGen Provides Genetic Insights From a Well‐Phenotyped Isolated Population.” Nature 613, no. 7944: 508–518. 36653562 10.1038/s41586-022-05473-8 PMC9849126

[brb370653-bib-0026] Lawlor, D. A. , R. M. Harbord , A. Tybjaerg‐Hansen , et al. 2011. “Using Genetic Loci to Understand the Relationship Between Adiposity and Psychological Distress: A Mendelian Randomization Study in the Copenhagen General Population Study of 53,221 Adults.” Journal of Internal Medicine 269, no. 5: 525–537. 21210875 10.1111/j.1365-2796.2011.02343.x

[brb370653-bib-0027] Locke, A. E. , B. Kahali , S. I. Berndt , et al. 2015. “Genetic Studies of Body Mass Index Yield New Insights for Obesity Biology.” Nature 518, no. 7538: 197–206. 25673413 10.1038/nature14177 PMC4382211

[brb370653-bib-0028] Luo, G. , Y. Li , C. Yao , M. Li , J. Li , and X. Zhang . 2023. “Prevalence of Overweight and Obesity in Patients With Major Depressive Disorder With Anxiety: Mediating Role of Thyroid Hormones and Metabolic Parameters.” Journal of Affective Disorders 335: 298–304. 37201896 10.1016/j.jad.2023.05.008

[brb370653-bib-0029] Mulugeta, A. , A. Zhou , K. S. Vimaleswaran , C. Dickson , and E. Hyppönen . 2019. “Depression Increases the Genetic Susceptibility to High Body Mass Index: Evidence From UK Biobank.” Depression and Anxiety 36, no. 12: 1154–1162. 31609059 10.1002/da.22963

[brb370653-bib-0030] Ogrodnik, M. , Y. Zhu , L. G. P. Langhi , et al. 2019. “Obesity‐Induced Cellular Senescence Drives Anxiety and Impairs Neurogenesis.” Cell Metabolism 29, no. 5: 1061–1077.e8. 30612898 10.1016/j.cmet.2018.12.008 PMC6509403

[brb370653-bib-0031] Panayotis, N. , P. A. Freund , L. Marvaldi , et al. 2021. “β‐sitosterol Reduces Anxiety and Synergizes With Established Anxiolytic Drugs in Mice.” Cell Reports Medicine 2, no. 5: 100281. 34095883 10.1016/j.xcrm.2021.100281 PMC8149471

[brb370653-bib-0032] Pedersen, B. K. , and B. Saltin . 2015. “Exercise as Medicine—Evidence for Prescribing Exercise as Therapy in 26 Different Chronic Diseases.” Supplement, Scandinavian Journal of Medicine & Science in Sports 25, no. S3: 1–72. 10.1111/sms.12581 26606383

[brb370653-bib-0033] Penninx, B. W. , D. S. Pine , E. A. Holmes , and A. Reif . 2021. “Anxiety Disorders.” Lancet 397, no. 10277: 914–927. 33581801 10.1016/S0140-6736(21)00359-7 PMC9248771

[brb370653-bib-0034] Puhl, R. M. , and C. A. Heuer . 2009. “The Stigma of Obesity: A Review and Update.” Obesity 17, no. 5: 941–964. 19165161 10.1038/oby.2008.636

[brb370653-bib-0035] Rajan, T. M. , and V. Menon . 2017. “Psychiatric Disorders and Obesity: A Review of Association Studies.” Journal of Postgraduate Medicine 63, no. 3: 182–190. 28695871 10.4103/jpgm.JPGM_712_16 PMC5525483

[brb370653-bib-0036] Sakayori, N. , H. Tokuda , K. Yoshizaki , et al. 2016. “Maternal Nutritional Imbalance Between Linoleic Acid and Alpha‐Linolenic Acid Increases Offspring's Anxious Behavior With a Sex‐Dependent Manner in Mice.” Tohoku Journal of Experimental Medicine 240, no. 1: 31–37. 27558477 10.1620/tjem.240.31

[brb370653-bib-0037] Samodien, E. , and N. Chellan . 2021. “Hypothalamic Neurogenesis and Its Implications for Obesity‐Induced Anxiety Disorders.” Frontiers in Neuroendocrinology 60: 100871. 32976907 10.1016/j.yfrne.2020.100871

[brb370653-bib-0038] Santiago Santana, J. M. , J. D. Vega‐Torres , P. Ontiveros‐Angel , et al. 2021. “Oxidative Stress and Neuroinflammation in a Rat Model of Co‐Morbid Obesity and Psychogenic Stress.” Behavioural Brain Research 400: 112995. 33301815 10.1016/j.bbr.2020.112995 PMC8713435

[brb370653-bib-0039] Sarwer, D. B. , and H. M. Polonsky . 2016. “The Psychosocial Burden of Obesity.” Endocrinology and Metabolism Clinics of North America 45, no. 3: 677–688. 27519139 10.1016/j.ecl.2016.04.016 PMC6052856

[brb370653-bib-0040] Simon, G. E. , M. Von Korff , K. Saunders , et al. 2006. “Association Between Obesity and Psychiatric Disorders in the US Adult Population.” Archives of General Psychiatry 63, no. 7: 824. 16818872 10.1001/archpsyc.63.7.824 PMC1913935

[brb370653-bib-0041] Skrivankova, V. W. , R. C. Richmond, B. A. R. Woolf, et al. 2021. “Strengthening the Reporting of Observational Studies in Epidemiology Using Mendelian Randomization: The STROBE‐MR Statement.” JAMA 326, no. 16: 1614. 34698778 10.1001/jama.2021.18236

[brb370653-bib-0042] Ströhle, A. , J. Gensichen , and K. Domschke . 2018. “The Diagnosis and Treatment of Anxiety Disorders.” Deutsches Ärzteblatt International 155, no. 37: 611–620. 30282583 10.3238/arztebl.2018.0611 PMC6206399

[brb370653-bib-0043] Szuhany, K. L. , and N. M. Simon . 2022. “Anxiety Disorders: A Review.” JAMA 328, no. 24: 2431. 36573969 10.1001/jama.2022.22744

[brb370653-bib-0044] Wang, R.‐Z. , Y. He , Y.‐T. Deng, et al. 2024. “Body Weight in Neurological and Psychiatric Disorders: A Large Prospective Cohort Study.” Nature Mental Health 2, no. 1: 41–51.

[brb370653-bib-0045] Wang, J. , X. Ran , J. Ye, et al. 2022. “Obesity‐Associated Anxiety Is Prevalent Among College Students and Alleviated by Calorie Restriction.” Nutrients 14, no. 17: 3518. 36079775 10.3390/nu14173518 PMC9460559

[brb370653-bib-0046] Wu, S. , C. Gennings , R. J. Wright , et al. 2018. “Prenatal Stress, Methylation in Inflammation‐Related Genes, and Adiposity Measures in Early Childhood: The Programming Research in Obesity, Growth Environment and Social Stress Cohort Study.” Psychosomatic Medicine 80, no. 1: 34–41. 28787364 10.1097/PSY.0000000000000517 PMC5741481

[brb370653-bib-0047] Xia, G. , Y. Han , F. Meng , et al. 2021. “Reciprocal Control of Obesity and Anxiety‐Depressive Disorder via a GABA and Serotonin Neural Circuit.” Molecular Psychiatry 26, no. 7: 2837–2853. 33767348 10.1038/s41380-021-01053-w PMC8505263

[brb370653-bib-0048] Xiao, G. , Q. He , L. Liu, et al. 2022. “Causality of Genetically Determined Metabolites on Anxiety Disorders: A Two‐Sample Mendelian Randomization Study.” Journal of Translational Medicine 20, no. 1: 475. 36266699 10.1186/s12967-022-03691-2 PMC9583573

[brb370653-bib-0049] Zhao, G. , E. S. Ford , S. Dhingra , C. Li , T. W. Strine , and A. H. Mokdad . 2009. “Depression and Anxiety Among US Adults: Associations With Body Mass Index.” International Journal of Obesity 33, no. 2: 257–266. 19125163 10.1038/ijo.2008.268

