# Supplementary material for: A Causal Inference Study of Circulating Metabolites Mediating the Effect of Obesity‐Related Indicators on the Incidence of Anxiety Disorders
Source: Brain Behav. 2025 Jul 7;15(7):e70653. doi: 10.1002/brb3.70653 (PMC12230357; doi:10.1002/brb3.70653)

Exposure	method	nSNPs	OR95CI	pvalue
Cholesterol to total lipids ratio in medium VLDL	Inverse variance weighted	62	0.909 (0.830, 0.996)	0.042
Cholesterol to total lipids ratio in small VLDL	Inverse variance weighted	51	0.915 (0.848, 0.987)	0.022
Cholesterol to total lipids ratio in very small VLDL	Inverse variance weighted	59	0.923 (0.853, 0.999)	0.048
Cholesteryl esters to total lipids ratio in medium VLDL	Inverse variance weighted	60	0.901 (0.816, 0.994)	0.038
Degree of unsaturation	Inverse variance weighted	35	0.936 (0.880, 0.995)	0.035
Free cholesterol to total lipids ratio in medium VLDL	Inverse variance weighted	51	0.911 (0.837, 0.992)	0.032
Free cholesterol to total lipids ratio in very small VLDL	Inverse variance weighted	40	0.903 (0.829, 0.984)	0.02
Phenylalanine	Inverse variance weighted	6	1.227 (1.001, 1.504)	0.049
Ratio of linoleic acid to total fatty acids	Inverse variance weighted	28	1.122 (1.016, 1.238)	0.023
Triglycerides to total lipids ratio in medium VLDL	Inverse variance weighted	55	1.104 (1.005, 1.214)	0.039
Triglycerides to total lipids ratio in small VLDL	Inverse variance weighted	53	1.102 (1.018, 1.194)	0.016
Triglycerides to total lipids ratio in very small VLDL	Inverse variance weighted	60	1.110 (1.025, 1.201)	0.0099

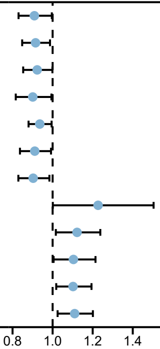

Supplement: Supplementary file 1 — Supplementary Figure: brb370653‐sup‐0001‐FigureS1.pdf [file BRB3-15-e70653-s011.pdf]
