# Supplementary figures and images for: A Causal Inference Study of Circulating Metabolites Mediating the Effect of Obesity‐Related Indicators on the Incidence of Anxiety Disorders
Source: Brain Behav. 2025 Jul 7;15(7):e70653. doi: 10.1002/brb3.70653 (PMC12230357; doi:10.1002/brb3.70653)

**A**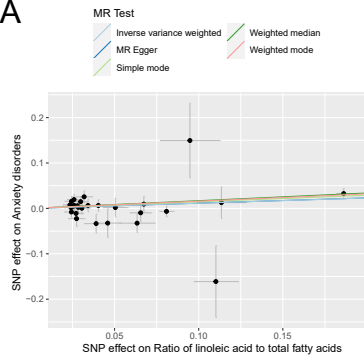**B**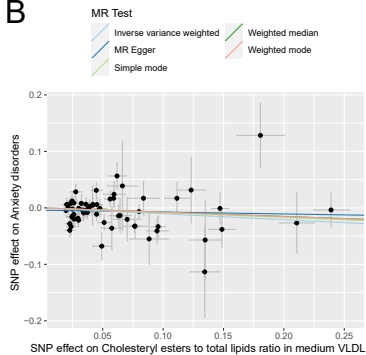**C**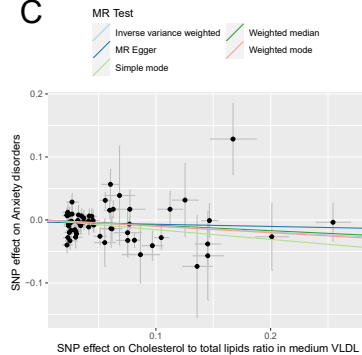**D**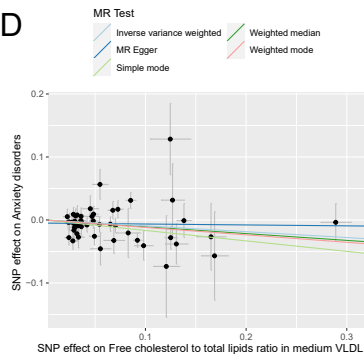**E**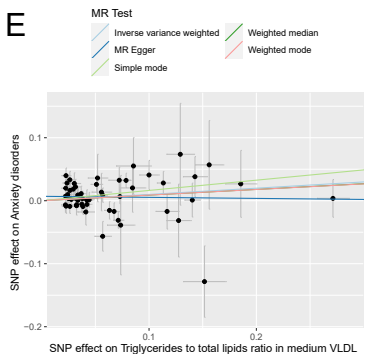**F**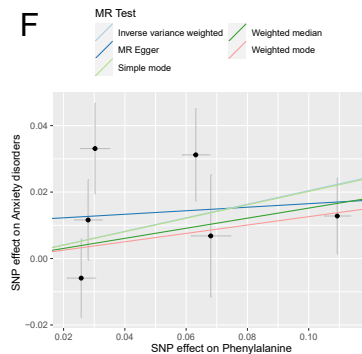**G**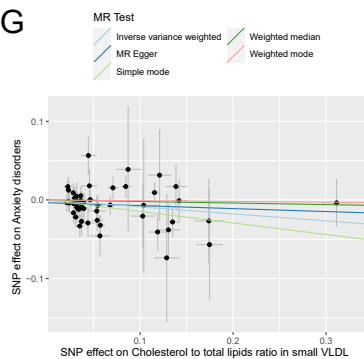**H**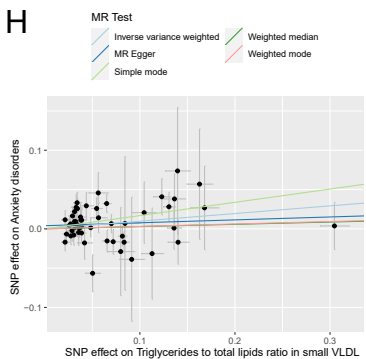**I**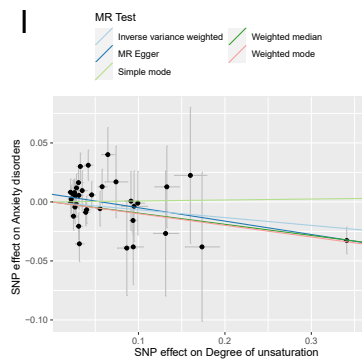**J**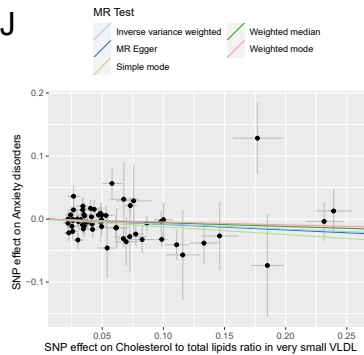**K**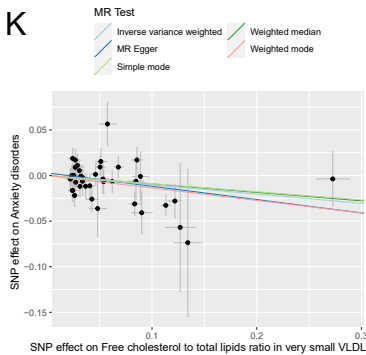**L**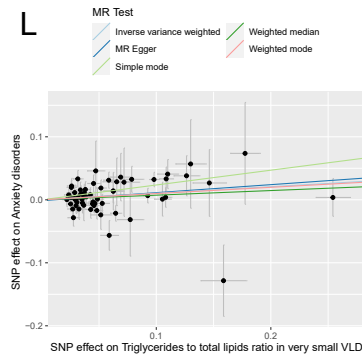

Supplement: Supplementary file 2 — Supplementary Figure: brb370653‐sup‐0002‐FigureS2.pdf [file BRB3-15-e70653-s014.pdf]

A

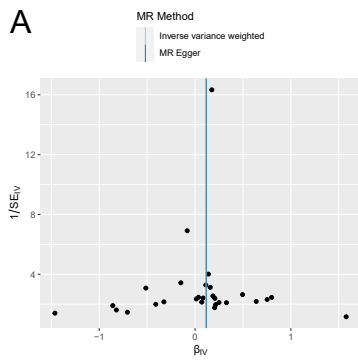

B

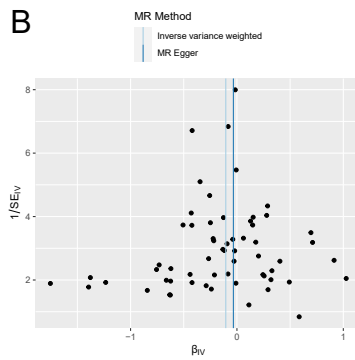

C

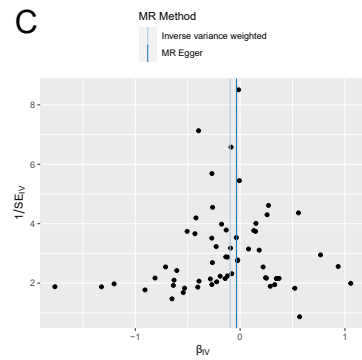

D

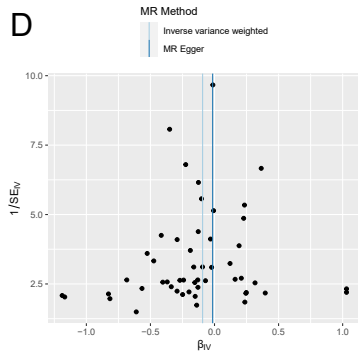

E

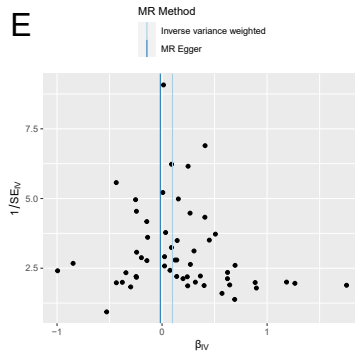

F

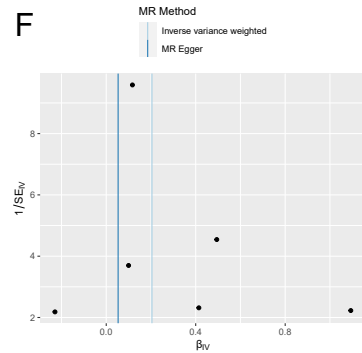

G

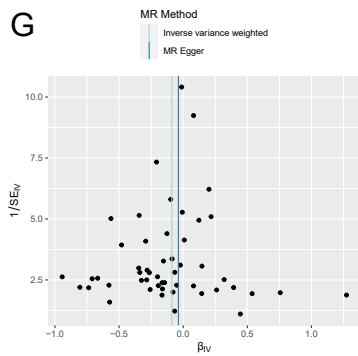

H

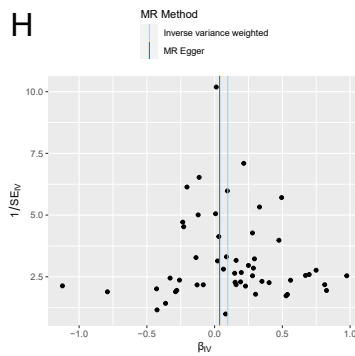

I

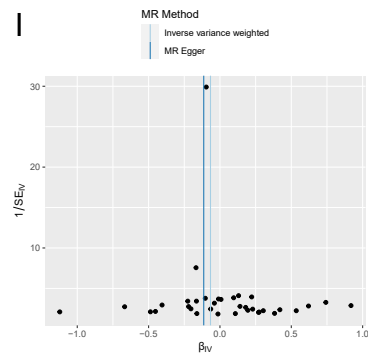

J

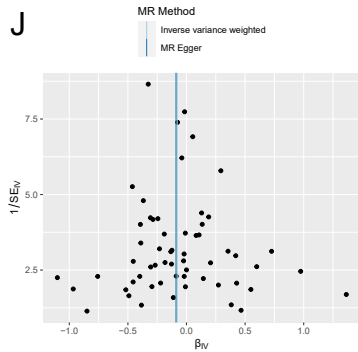

K

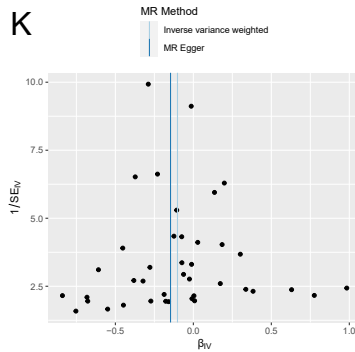

L

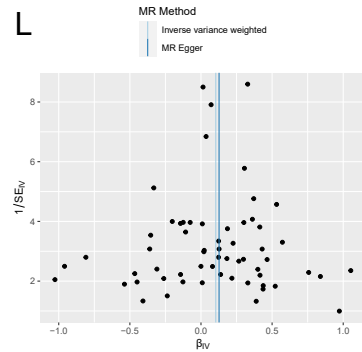

Supplement: Supplementary file 3 — Supplementary Figure: brb370653‐sup‐0003‐FigureS3.pdf [file BRB3-15-e70653-s017.pdf]

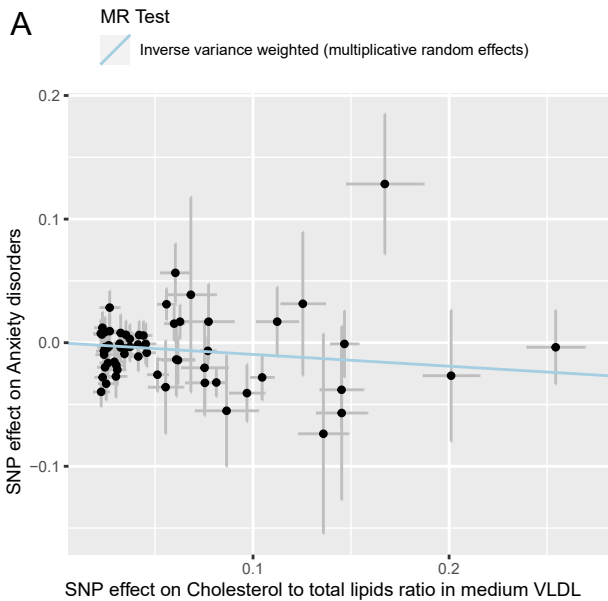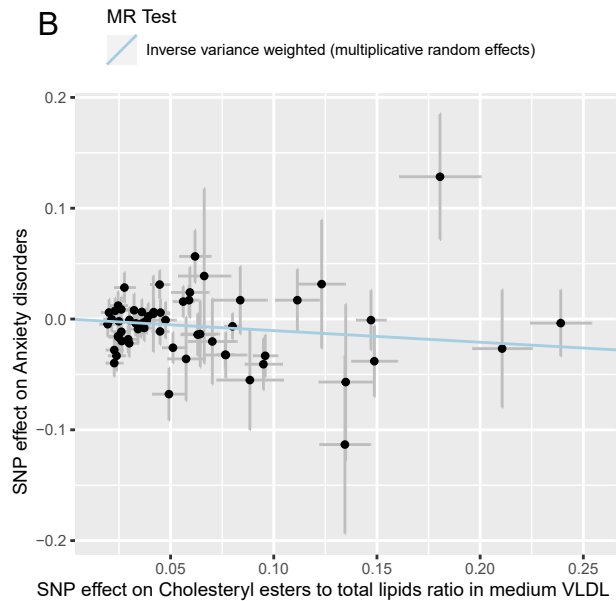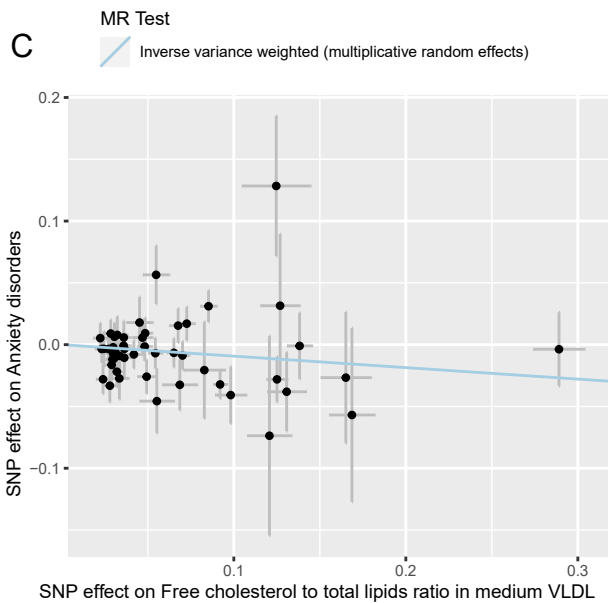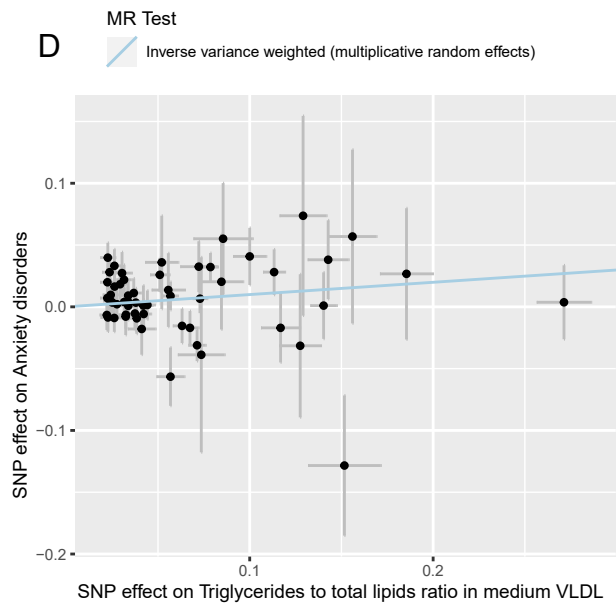

Supplement: Supplementary file 4 — Supplementary Figure: brb370653‐sup‐0004‐FigureS4.pdf [file BRB3-15-e70653-s015.pdf]
