# Supplementary material for: A Causal Inference Study of Circulating Metabolites Mediating the Effect of Obesity‐Related Indicators on the Incidence of Anxiety Disorders
Source: Brain Behav. 2025 Jul 7;15(7):e70653. doi: 10.1002/brb3.70653 (PMC12230357; doi:10.1002/brb3.70653)
Supplement: Supplementary file 5 — Supplementary Figure: brb370653‐sup‐0005‐Table1.docx [file BRB3-15-e70653-s004.docx]

Supplementary Table 1 Selection of instrumental variables for Obesity-related index, Circulating metabolites and Anxiety disorders

| Exposure | Number of SNPs | Median of F | Minimum of F | Maximum of F |
| --- | --- | --- | --- | --- |
| **Obesity-related index** | | | | |
| Body fat percentage | 217 | 41.20593213 | 433.5838458 | 30.01459245 |
| Obesity and other hyperalimentation | 8 | 35.1192447 | 50.44758174 | 29.99326238 |
| **Circulating metabolites** | | | | |
| Cholesterol to total lipids ratio in medium VLDL | 62 | 59.19020733 | 414.1871004 | 26.84561823 |
| Cholesterol to total lipids ratio in small VLDL | 51 | 61.44737987 | 723.8836551 | 27.87040378 |
| Cholesterol to total lipids ratio in very small VLDL | 59 | 71.62712333 | 575.4448816 | 29.6422262 |
| Cholesteryl esters to total lipids ratio in medium VLDL | 60 | 61.35013306 | 419.1808157 | 24.69188226 |
| Degree of unsaturation | 35 | 59.28040573 | 6916.69434 | 27.86859854 |
| Free cholesterol to total lipids ratio in medium VLDL | 51 | 59.45645276 | 497.0084309 | 28.69053022 |
| Free cholesterol to total lipids ratio in very small VLDL | 40 | 59.20943758 | 694.3801576 | 28.40598397 |
| Phenylalanine | 6 | 77.3687287 | 664.4838404 | 31.57460202 |
| Ratio of linoleic acid to total fatty acids | 28 | 42.16665954 | 1940.437443 | 28.64630522 |
| Triglycerides to total lipids ratio in medium VLDL | 55 | 62.15059846 | 376.5212805 | 27.93641348 |
| Triglycerides to total lipids ratio in small VLDL | 53 | 58.68996806 | 453.1075541 | 26.75147668 |
| Triglycerides to total lipids ratio in very small VLDL | 60 | 63.65007685 | 565.0957569 | 28.12156878 |

SNPs，Single Nucleotide Polymorphisms；F，F statistics.
