# Supplementary material for: A Causal Inference Study of Circulating Metabolites Mediating the Effect of Obesity‐Related Indicators on the Incidence of Anxiety Disorders
Source: Brain Behav. 2025 Jul 7;15(7):e70653. doi: 10.1002/brb3.70653 (PMC12230357; doi:10.1002/brb3.70653)
Supplement: Supplementary file 6 — Supplementary Figure: brb370653‐sup‐0006‐Table2.docx [file BRB3-15-e70653-s007.docx]

Supplementary Table 2 Mendelian randomization analysis heterogeneity test for the association between Obesity-related index and Anxiety disorders.

| Exposure | Q | Q df | Cochran Q p-value | I^2^ (%) |
| --- | --- | --- | --- | --- |
| Obesity and other hyperalimentation | 8.653398807 | 7 | 0.27850742 | 19.11% |
| Body fat percentage | 262.2823868 | 216 | 0.017147184 | 17.65% |

Q，Cochran's Q test statistic；Q df，degrees of freedom for the Q test；I^2^ statistic reflects the proportion of heterogeneity attributed to instrumental variables in the total variability。
