# Supplementary material for: A Causal Inference Study of Circulating Metabolites Mediating the Effect of Obesity‐Related Indicators on the Incidence of Anxiety Disorders
Source: Brain Behav. 2025 Jul 7;15(7):e70653. doi: 10.1002/brb3.70653 (PMC12230357; doi:10.1002/brb3.70653)
Supplement: Supplementary file 7 — Supplementary Figure: brb370653‐sup‐0007‐Table3.docx [file BRB3-15-e70653-s010.docx]

Supplementary Table 3 Inverse variance weighted random-effects model analysis of the association between Obesity-related index and Anxiety disorders.

| Exposure | Number of SNPs | Beta | Standard error | P value |
| --- | --- | --- | --- | --- |
| Obesity and other hyperalimentation | Inverse variance weighted (multiplicative random effects) | 8 | -0.103363923 | 0.043605497 |
| Body fat percentage | Inverse variance weighted (multiplicative random effects) | 217 | 0.133439736 | 0.062490584 |

SNPs：Single Nucleotide Polymorphisms。
