# Supplementary material for: A Causal Inference Study of Circulating Metabolites Mediating the Effect of Obesity‐Related Indicators on the Incidence of Anxiety Disorders
Source: Brain Behav. 2025 Jul 7;15(7):e70653. doi: 10.1002/brb3.70653 (PMC12230357; doi:10.1002/brb3.70653)
Supplement: Supplementary file 8 — Supplementary Figure: brb370653‐sup‐0008‐Table4.docx [file BRB3-15-e70653-s002.docx]

Supplementary Table 4 Mendelian randomization analysis of horizontal pleiotropy for the association between Obesity-related index and Anxiety disorders

| Exposure | MR-Egger intercept | Standard error | P value |
| --- | --- | --- | --- |
| Obesity and other hyperalimentation | 0.009874474 | 0.022618876 | 0.677703012 |
| Body fat percentage | 0.006326814 | 0.003407059 | 0.064682895 |
