# Supplementary material for: A Causal Inference Study of Circulating Metabolites Mediating the Effect of Obesity‐Related Indicators on the Incidence of Anxiety Disorders
Source: Brain Behav. 2025 Jul 7;15(7):e70653. doi: 10.1002/brb3.70653 (PMC12230357; doi:10.1002/brb3.70653)
Supplement: Supplementary file 11 — Supplementary Figure: brb370653‐sup‐00011‐Table6.docx [file BRB3-15-e70653-s003.docx]

Supplementary Table 6 Results of reverse causal Mendelian randomization analysis of Obesity-related index on Anxiety disorders

| Exposure | outcome | Number of SNPs | b | Standard error | P value |
| --- | --- | --- | --- | --- | --- |
| Anxiety disorders | Obesity and other hyperalimentation | 19 | 0.083346947 | 0.069995298 | 0.233751489 |
| Anxiety disorders | Body fat percentage | 19 | -0.048078731 | 0.19216496 | 0.805269788 |

SNPs：Single Nucleotide Polymorphisms。
