# Supplementary material for: A Causal Inference Study of Circulating Metabolites Mediating the Effect of Obesity‐Related Indicators on the Incidence of Anxiety Disorders
Source: Brain Behav. 2025 Jul 7;15(7):e70653. doi: 10.1002/brb3.70653 (PMC12230357; doi:10.1002/brb3.70653)
Supplement: Supplementary file 12 — Supplementary Figure: brb370653‐sup‐00012‐Table7.docx [file BRB3-15-e70653-s001.docx]

Supplementary Table 7 Mendelian randomization analysis heterogeneity test for the association between Circulating metabolites and Anxiety disorders

| Exposure | Q | Q df | Cochran Q p-value | I^2^ (%) |
| --- | --- | --- | --- | --- |
| Ratio of linoleic acid to total fatty acids | 32.11188618 | 27 | 0.227965766 | 15.92% |
| Cholesterol to total lipids ratio in medium VLDL | 90.25269513 | 61 | 0.008828716 | 32.41% |
| Cholesteryl esters to total lipids ratio in medium VLDL | 96.60125164 | 59 | 0.001459953 | 38.92% |
| Free cholesterol to total lipids ratio in medium VLDL | 68.04155134 | 50 | 0.045634583 | 26.52% |
| Triglycerides to total lipids ratio in medium VLDL | 82.9308666 | 54 | 0.006898817 | 34.89% |
| Phenylalanine | 7.628042128 | 5 | 0.177961503 | 34.45% |
| Cholesterol to total lipids ratio in small VLDL | 54.95843941 | 50 | 0.292348146 | 9.02% |
| Triglycerides to total lipids ratio in small VLDL | 59.80434147 | 52 | 0.213398705 | 13.05% |
| Degree of unsaturation | 40.70747944 | 34 | 0.199043431 | 16.48% |
| Cholesterol to total lipids ratio in very small VLDL | 72.01337356 | 58 | 0.10208095 | 19.46% |
| Free cholesterol to total lipids ratio in very small VLDL | 47.16707975 | 39 | 0.173243138 | 17.32% |
| Triglycerides to total lipids ratio in very small VLDL | 73.22962415 | 59 | 0.10069158 | 19.43% |

Q，Cochran's Q test statistic；Q df，degrees of freedom for the Q test；I^2^ statistic reflects the proportion of heterogeneity attributed to instrumental variables in the total variability。
