# Supplementary material for: A Causal Inference Study of Circulating Metabolites Mediating the Effect of Obesity‐Related Indicators on the Incidence of Anxiety Disorders
Source: Brain Behav. 2025 Jul 7;15(7):e70653. doi: 10.1002/brb3.70653 (PMC12230357; doi:10.1002/brb3.70653)
Supplement: Supplementary file 13 — Supplementary Figure: brb370653‐sup‐00013‐Table8.docx [file BRB3-15-e70653-s019.docx]

Supplementary Table 8 Inverse variance weighted random-effects model analysis of the association between Circulating metabolites and Anxiety disorders.

| Exposure | Number of SNPs | Beta | Standard error | P value |
| --- | --- | --- | --- | --- |
| Ratio of linoleic acid to total fatty acids | 28 | 0.11476514 | 0.05053342 | 0.023142494 |
| Cholesterol to total lipids ratio in medium VLDL | 62 | -0.095052875 | 0.046645733 | 0.041573799 |
| Cholesteryl esters to total lipids ratio in medium VLDL | 60 | -0.104789943 | 0.050542285 | 0.038143221 |
| Free cholesterol to total lipids ratio in medium VLDL | 51 | -0.092906403 | 0.043449107 | 0.032493937 |
| Triglycerides to total lipids ratio in medium VLDL | 55 | 0.099350428 | 0.048250814 | 0.039490263 |
| Phenylalanine | 6 | 0.204332094 | 0.10390361 | 0.049234612 |
| Cholesterol to total lipids ratio in small VLDL | 51 | -0.088817774 | 0.038705561 | 0.021750141 |
| Triglycerides to total lipids ratio in small VLDL | 53 | 0.09743171 | 0.040566224 | 0.016314898 |
| Degree of unsaturation | 35 | -0.066300287 | 0.031412087 | 0.034801407 |
| Cholesterol to total lipids ratio in very small VLDL | 59 | -0.079696173 | 0.040232834 | 0.047605409 |
| Free cholesterol to total lipids ratio in very small VLDL | 40 | -0.101728695 | 0.043887761 | 0.020453205 |
| Triglycerides to total lipids ratio in very small VLDL | 60 | 0.104106422 | 0.040358762 | 0.009893639 |

SNPs：Single Nucleotide Polymorphisms。
