# Supplementary material for: A Causal Inference Study of Circulating Metabolites Mediating the Effect of Obesity‐Related Indicators on the Incidence of Anxiety Disorders
Source: Brain Behav. 2025 Jul 7;15(7):e70653. doi: 10.1002/brb3.70653 (PMC12230357; doi:10.1002/brb3.70653)
Supplement: Supplementary file 14 — Supplementary Figure: brb370653‐sup‐00014‐Table9.docx [file BRB3-15-e70653-s013.docx]

Supplementary Table 9 Mendelian randomization analysis of horizontal pleiotropy for the association between Circulating metabolites and Anxiety disorders

| Exposure | MR-Egger intercept | Standard error | P value |
| --- | --- | --- | --- |
| Ratio of linoleic acid to total fatty acids | -0.00012788 | 0.00443997 | 0.97724237 |
| Cholesterol to total lipids ratio in medium VLDL | -0.003404469 | 0.00411839 | 0.411710556 |
| Cholesteryl esters to total lipids ratio in medium VLDL | -0.004073412 | 0.004575362 | 0.376986817 |
| Free cholesterol to total lipids ratio in medium VLDL | -0.004950712 | 0.004319022 | 0.257255529 |
| Triglycerides to total lipids ratio in medium VLDL | 0.006973216 | 0.004350631 | 0.114921342 |
| Phenylalanine | 0.011189247 | 0.013049525 | 0.439535233 |
| Cholesterol to total lipids ratio in small VLDL | -0.00352501 | 0.003733583 | 0.349732519 |
| Triglycerides to total lipids ratio in small VLDL | 0.004000758 | 0.003989416 | 0.320671115 |
| Degree of unsaturation | 0.006459785 | 0.003241295 | 0.054588091 |
| Cholesterol to total lipids ratio in very small VLDL | 0.000609196 | 0.004142587 | 0.883605973 |
| Free cholesterol to total lipids ratio in very small VLDL | 0.002850327 | 0.004229145 | 0.504408188 |
| Triglycerides to total lipids ratio in very small VLDL | -0.001395148 | 0.004245215 | 0.743611388 |
