# Supplementary material for: A Causal Inference Study of Circulating Metabolites Mediating the Effect of Obesity‐Related Indicators on the Incidence of Anxiety Disorders
Source: Brain Behav. 2025 Jul 7;15(7):e70653. doi: 10.1002/brb3.70653 (PMC12230357; doi:10.1002/brb3.70653)
Supplement: Supplementary file 17 — Supplementary Figure: brb370653‐sup‐00017‐Table11.docx [file BRB3-15-e70653-s008.docx]

Supplementary Table 11 Results of reverse causal Mendelian randomization analysis of Circulating metabolites on Anxiety disorders

| Exposure | outcome | Number of SNPs | Beta | Standard error | P value |
| --- | --- | --- | --- | --- | --- |
| Anxiety disorders | Ratio of linoleic acid to total fatty acids | 19 | 0.003578969 | 0.015060537 | 0.812161199 |
| Anxiety disorders | Cholesterol to total lipids ratio in medium VLDL | 19 | -0.004419691 | 0.015681176 | 0.778060895 |
| Anxiety disorders | Cholesteryl esters to total lipids ratio in medium VLDL | 19 | -0.004497816 | 0.015407301 | 0.770342003 |
| Anxiety disorders | Free cholesterol to total lipids ratio in medium VLDL | 19 | -0.004107923 | 0.016323541 | 0.80130667 |
| Anxiety disorders | Triglycerides to total lipids ratio in medium VLDL | 19 | 0.004239496 | 0.015981515 | 0.790797656 |
| Anxiety disorders | Phenylalanine | 19 | 0.010476476 | 0.020882234 | 0.615883304 |
| Anxiety disorders | Cholesterol to total lipids ratio in small VLDL | 19 | -0.010886486 | 0.018134407 | 0.548291567 |
| Anxiety disorders | Triglycerides to total lipids ratio in small VLDL | 19 | 0.012433336 | 0.017989525 | 0.489475628 |
| Anxiety disorders | Degree of unsaturation | 19 | 0.02104406 | 0.016104406 | 0.191305502 |
| Anxiety disorders | Cholesterol to total lipids ratio in very small VLDL | 19 | -0.00982626 | 0.015121381 | 0.51580493 |
| Anxiety disorders | Free cholesterol to total lipids ratio in very small VLDL | 19 | -0.00836166 | 0.018752712 | 0.655676184 |

SNPs：Single Nucleotide Polymorphisms。
