# Supplementary material for: A Causal Inference Study of Circulating Metabolites Mediating the Effect of Obesity‐Related Indicators on the Incidence of Anxiety Disorders
Source: Brain Behav. 2025 Jul 7;15(7):e70653. doi: 10.1002/brb3.70653 (PMC12230357; doi:10.1002/brb3.70653)
Supplement: Supplementary file 18 — Supplementary Figure: brb370653‐sup‐00018‐Table12.docx [file BRB3-15-e70653-s012.docx]

Supplementary Table 12 Inverse variance weighted random-effects model analysis of Obesity-related index and Circulating metabolites

| Exposure | Outcome | Number of SNPs | Beta | Standard error | P value |
| --- | --- | --- | --- | --- | --- |
| Obesity and other hyperalimentation | Phenylalanine | 8 | 0.029576 | 0.014157 | 0.036694 |
| Body fat percentage | Ratio of linoleic acid to total fatty acids | 229 | -0.39205 | 0.040895 | 9.10E-22 |
| Body fat percentage | Cholesterol to total lipids ratio in medium VLDL | 229 | -0.29651 | 0.047152 | 3.21E-10 |
| Body fat percentage | Cholesteryl esters to total lipids ratio in medium VLDL | 229 | -0.2997 | 0.047427 | 2.63E-10 |
| Body fat percentage | Free cholesterol to total lipids ratio in medium VLDL | 229 | -0.27821 | 0.045449 | 9.27E-10 |
| Body fat percentage | Triglycerides to total lipids ratio in medium VLDL | 229 | 0.290216 | 0.047073 | 7.04E-10 |
| Body fat percentage | Phenylalanine | 229 | 0.168157 | 0.022529 | 8.39E-14 |
| Body fat percentage | Cholesterol to total lipids ratio in small VLDL | 229 | -0.21187 | 0.047304 | 7.50E-06 |
| Body fat percentage | Triglycerides to total lipids ratio in small VLDL | 229 | 0.238235 | 0.048981 | 1.15E-06 |
| Body fat percentage | Degree of unsaturation | 229 | -0.24353 | 0.031336 | 7.74E-15 |
| Body fat percentage | Cholesterol to total lipids ratio in very small VLDL | 229 | -0.33661 | 0.047535 | 1.43E-12 |
| Body fat percentage | Free cholesterol to total lipids ratio in very small VLDL | 229 | -0.1547 | 0.035146 | 1.07E-05 |
| Body fat percentage | Triglycerides to total lipids ratio in very small VLDL | 229 | 0.31157 | 0.047518 | 5.49E-11 |

SNP， single nucleotide polymorphism。
