# Supplementary material for: A Causal Inference Study of Circulating Metabolites Mediating the Effect of Obesity‐Related Indicators on the Incidence of Anxiety Disorders
Source: Brain Behav. 2025 Jul 7;15(7):e70653. doi: 10.1002/brb3.70653 (PMC12230357; doi:10.1002/brb3.70653)
Supplement: Supplementary file 19 — Supplementary Figure: brb370653‐sup‐00019‐Table13.docx [file BRB3-15-e70653-s016.docx]

Supplementary Table 13 Mendelian randomization analysis of Obesity-related index and Circulating metabolites Steiger directionality test

| Exposure | Outcome | SNP r^2^ exposure | SNP r^2^ outcome | Correct causal direction | Steiger P value |
| --- | --- | --- | --- | --- | --- |
| Obesity and other hyperalimentation | Phenylalanine | 0.002186121 | 0.000197413 | TRUE | 2.49E-19 |
| Body fat percentage | Ratio of linoleic acid to total fatty acids | 0.040539451 | 0.013119104 | TRUE | 2.41E-149 |
| Body fat percentage | Cholesterol to total lipids ratio in medium VLDL | 0.040539451 | 0.01576396 | TRUE | 9.50E-115 |
| Body fat percentage | Cholesteryl esters to total lipids ratio in medium VLDL | 0.040539451 | 0.016109942 | TRUE | 9.41E-111 |
| Body fat percentage | Free cholesterol to total lipids ratio in medium VLDL | 0.040539451 | 0.014161476 | TRUE | 7.95E-135 |
| Body fat percentage | Triglycerides to total lipids ratio in medium VLDL | 0.040539451 | 0.015419998 | TRUE | 7.78E-119 |
| Body fat percentage | Phenylalanine | 0.040539451 | 0.003279283 | TRUE | 0 |
| Body fat percentage | Cholesterol to total lipids ratio in small VLDL | 0.040539451 | 0.013568901 | TRUE | 5.80E-143 |
| Body fat percentage | Triglycerides to total lipids ratio in small VLDL | 0.040539451 | 0.014950095 | TRUE | 1.31E-124 |
| Body fat percentage | Degree of unsaturation | 0.040539451 | 0.007778425 | TRUE | 1.70E-250 |
| Body fat percentage | Cholesterol to total lipids ratio in very small VLDL | 0.040539451 | 0.016306631 | TRUE | 1.57E-108 |
| Body fat percentage | Free cholesterol to total lipids ratio in very small VLDL | 0.040539451 | 0.007319879 | TRUE | 3.57E-262 |
| Body fat percentage | Triglycerides to total lipids ratio in very small VLDL | 0.040539451 | 0.015796266 | TRUE | 2.27E-114 |

SNP, single nucleotide polymorphism；r^2^, variance explained rate.
